# mPTP opening caused by Cdk5 loss is due to increased mitochondrial Ca^2+^ uptake

**DOI:** 10.1038/s41388-020-1188-5

**Published:** 2020-02-05

**Authors:** Saranya NavaneethaKrishnan, Jesusa L. Rosales, Ki-Young Lee

**Affiliations:** Departments of Cell Biology and Anatomy, Arnie Charbonneau Cancer Institute and Alberta Children’s Hospital Research Institute, Cumming School of Medicine, University of Calgary, Calgary, AB Canada

**Keywords:** Breast cancer, Cell signalling

## Abstract

We previously demonstrated that loss of Cdk5 in breast cancer cells promotes ROS-mediated cell death by inducing mitochondrial permeability transition pore (mPTP) opening (Oncogene 37, 1788–1804). However, the molecular mechanism by which Cdk5 loss causes mPTP opening remains to be investigated. Using primary mouse embryonic fibroblasts (MEFs) isolated from *Cdk5*^*−/−*^ mouse embryos, we show that absence of Cdk5 causes a significant increase in both mPTP opening and mitochondrial Ca^2+^ level. Analysis of subcellular fractions of MEFs demonstrates that Cdk5 localizes in the mitochondria-associated endoplasmic reticulum (ER) membrane (MAM) and Cdk5 loss in MAMs causes increased ER-mitochondria tethering, a process required for Ca^2+^ transfer from the ER to the mitochondria. Loss of Cdk5 also causes increased ATP-mediated mitochondrial Ca^2+^ uptake from the ER. Inhibition of ER Ca^2+^ release or mitochondrial Ca^2+^ uptake in *Cdk5*^*−/−*^ MEFs prevents mPTP opening, indicating that mPTP opening in *Cdk5*^*−/−*^ MEFs is due to increased Ca^2+^ transfer from the ER to the mitochondria. Altogether, our findings suggest that Cdk5 in MAMs regulates mitochondrial Ca^2+^ homeostasis that is disturbed upon Cdk5 loss, which leads to mPTP opening.

## Introduction

Cyclin dependent kinase 5 (Cdk5) is a small proline-directed serine/threonine kinase that serves various cell functions such as in the regulation of oxidative stress [1, 2], mitochondrial functions [3] and apoptosis [4, 5]. We previously reported that loss of Cdk5 promotes mitochondrial permeability transition pore (mPTP)-mediated apoptosis in breast cancer cells [6]. We found that Cdk5 loss induces mPTP opening, which leads to increased cellular levels of reactive oxygen species (ROS) that subsequently cause increased susceptibility of breast cancer cells to apoptosis. However, the fundamental mechanism by which loss of Cdk5 regulates mPTP opening is unclear.

Mitochondria are cell organelles that execute a number of key cellular functions, including in energy conversion, intermediate cellular metabolism, cell differentiation, immune response, calcium signaling and homeostasis, and cell death. While the outer mitochondrial membrane (OMM) is highly permeable to Ca^2+^, the inner mitochondrial membrane (IMM) contains the mitochondrial calcium uniporter (MCU) complex that regulates mitochondrial calcium ([Ca^2+^]_mt_) influx [7]. As MCU has low affinity to Ca^2+^ with K_d_ of ~10 μM, high Ca^2+^ concentration is needed for MCU activity [8]. Mitochondrial uptake of Ca^2+^ through MCU channels is facilitated by the close proximity of the mitochondria to the endoplasmic reticulum (ER) [9, 10]. A common mechanism by which mitochondria communicate with the ER is through the interface between these two organelles, which is designated as the mitochondria-associated ER membrane (MAM) [11]. MAM regulates transport of lipids, calcium and other metabolites from the ER to the mitochondria [12, 13], and is involved in the regulation of mitochondrial dynamics [14, 15], formation of autophagosomes and cell survival [16, 17]. MAM consists of inositol 1,4,5-trisphosphate receptors (IP3Rs), voltage-dependent anion channels (VDACs), glucose-regulated protein 75 kDa (Grp75), glucose- regulated protein 78 kDa (Grp78) [18], mitofusin 2 (Mfn2), long-chain fatty acid-CoA ligase 4 (FACL-4), phosphofurin acidic cluster sorting protein 2 (PACS-2), B-cell receptor-associated protein 31 (Bap31) and mitochondrial fission 1 protein (Fis1) [19], and contains a high concentration of calcium [20]. Once Ca^2+^ is released from the ER to the cytoplasm via the IP3R channels, part of the released Ca^2+^ is taken up by the mitochondria [21] through the VDACs in the OMM, and the MCU channels in the IMM [10, 22]. Several studies show rapid Ca^2+^ mobilization from IP3-gated channels to nearby mitochondria, resulting in increased [Ca^2+^]_mt_ concentration [23–25]. The ER is closely tethered to approximately 5–20% of mitochondrial surface within a 10–30 nm space [26, 27]. This distinct structure is crucial for intracellular calcium homeostasis. Thus, disruption of MAM causes deregulation of calcium mobilization and disruption of [Ca^2+^]_mt_ homeostasis.

Under physiological conditions, a small rise in mitochondrial Ca^2+^ concentration leads to increased mitochondrial respiratory chain activity and ATP synthesis [28, 29]. However, pathological mitochondrial calcium overload is associated with increased generation of ROS, mitochondrial depolarization and increased or prolonged mPTP opening [28–30]. The voltage-dependent, high-conductance mPTP channel controls permeabilization of the IMM. While transient opening of mPTP is thought to be a calcium efflux channel in the mitochondria under normal conditions [31, 32], prolonged mPTP opening causes mitochondrial swelling and release of cytochrome C and other intermembrane space (IMS) proteins, leading to activation of caspase-mediated apoptosis [33, 34].

In this study, we used *Cdk5*^*−/−*^ mouse embryonic fibroblasts (MEFs) to investigate how Cdk5 loss induces mPTP opening. We demonstrate that loss of Cdk5 alters ER-mitochondria tethering, increasing mitochondrial Ca^2+^ uptake from the ER. We propose that Cdk5 loss alters mitochondrial Ca^2+^ homeostasis, causing mPTP opening.

## Results

### Cdk5 loss in primary MEFs induces mPTP opening

**Fig. 1:**
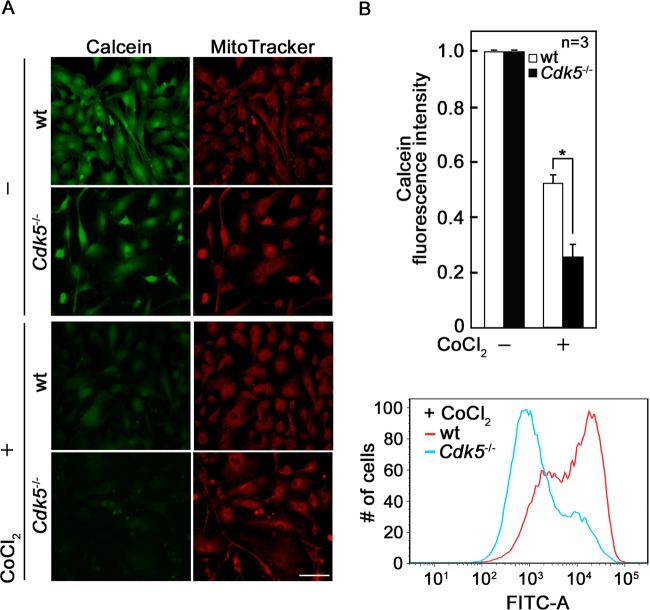
Absence of Cdk5 induces mPTP opening. **a** Wt and *Cdk5*^*−/−*^ MEFs loaded with calcein-AM (1 µM) and mitotracker red (200 nM) were treated with or without CoCl_2_ and analyzed by fluorescence microscopy. Images were acquired using an Olympus 1 × 71 microscope at ×160 magnification. Scale bar = 100 µm. Data represent one of three (*n* = 3) independent experiments showing a similar staining pattern. **b** Quantitative analysis of calcein fluorescence intensity in the presence and absence of CoCl_2_ in wt and *Cdk5*^*−/−*^ MEFs as determined by flow cytometry. Values for wt and *Cdk5*^*−/−*^ MEFs loaded with calcein-AM alone were normalized to 1.0. The relative calcein fluorescence intensity in MEFs treated with CoCl_2_ were then calculated. Values are means ± SEM from three (*n* = 3) independent experiments. For each experiment, 1 × 10^4^ cells were used for flow cytometry analysis. *p < 0.05 by unpaired Student’s *t* test. **c** Wt and *Cdk5*^*−/−*^ MEFs loaded with calcein-AM were treated with CoCl_2_ and subjected to flow cytometry analysis. Data represent one of three (*n* = 3) independent experiments showing similar results.

Previously, we demonstrated that knocking down Cdk5 by siRNA in breast cancer cells causes mPTP opening and subsequent ROS increase, which promotes cell death [6]. To further characterize the molecular and cellular mechanisms that lead to mPTP opening upon Cdk5 loss, we utilized primary MEFs isolated from wt and *Cdk5*^*−/−*^ mouse embryos as knockout of the *Cdk5* gene in mice is associated with perinatal lethality [39]. Initially, we assessed mPTP opening in *Cdk5*^*−/−*^ MEFs by calcein-AM staining followed by treatment with CoCl_2_. Calcein-AM is a cell permeable fluorophore that diffuses and gets trapped in all subcellular compartments, including mitochondria [40]. Treatment with cobalt (Co^2+^) quenches calcein fluorescence in all subcellular compartments except the mitochondrial matrix which is enclosed by a Co^2+^ impermeable inner mitochondrial membrane when mPTP is closed. Thus, the ability of Co^2+^ to quench mitochondrial calcein fluorescence only when mPTP is open allows determination of open vs closed status of mPTP in the cell [40]. As shown in Fig. 1a, fluorescence microscopy of wt and *Cdk5*^*−/−*^ MEFs following calcein staining without CoCl_2_ treatment showed strong and similar fluorescence intensity, indicating equivalent intracellular calcein-AM loading. However, upon treatment with CoCl_2_, *Cdk5*^*−/−*^ MEFs exhibited less calcein fluorescence intensity compared with wt, indicating greater quenching of mitochondrial calcein fluorescence and thus increased mPTP opening in *Cdk5*^*−/−*^ MEFs compared with wt. Consistent with these observations, flow cytometry analyses of CoCl_2_-treated cells pre-stained with calcein (Fig. 1b, top and bottom panels) showed that *Cdk5*^*−/−*^ MEFs have reduced (*p* < 0.05) calcein fluorescence intensity compared to wt (0.25 vs 0.52). The clear decline in calcein-stained population of *Cdk5*^*−/−*^ MEFs further indicates greater mPTP opening in these cells compared with wt.

### Loss of Cdk5 in MEFs causes increased level of [Ca^2+^]_mt_

**Fig. 2:**
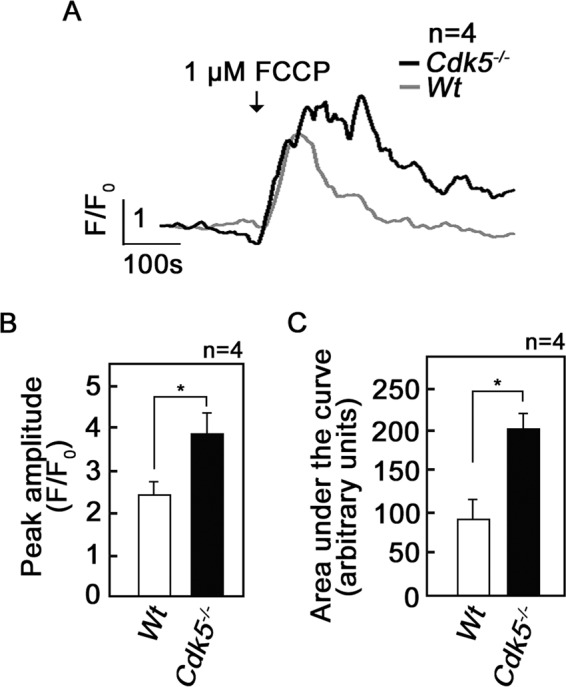
Loss of Cdk5 causes increased mitochondrial Ca^2+^ level. MEFs were isolated from wt and *Cdk5*^*−/−*^ embryos from *Cdk5*^+/−^ pregnant female mice. Cells loaded with Fluo-4 AM (5 µM) were subjected to single-cell Ca^2+^ imaging in Ca^2+^ free buffer. Carbonyl cyanide-p-trifluoromethoxyphenylhydrazone (FCCP, 1 µM) was added where indicated. **a** FCCP-induced Ca^2+^ flux to the cytoplasm is greater in *Cdk5*^*−/−*^ MEFs than in wt MEFs. Mean values of Ca^2+^ signals from 15 randomly selected cells from each genotype are shown. Data represent results from one of four independent experiments (*n* = 4) showing a similar staining patterns. Analysis of results from all four independent experiments, each with data from 15 randomly selected cells from wt and *Cdk5*^*−/−*^ MEFs, revealed that: **b** the peak amplitudes (*F*/*F*_0_) of Ca^2+^ response to FCCP is higher in *Cdk5*^*−/−*^ MEFs than in wt, and (**c**) the integrated Ca^2+^ signals (area under the curve from 180 to 600 s) in response to FCCP is also higher in Cdk5^*−/−*^ MEFs than in wt, indicating greater Ca^2^flux to the cytoplasm due to increased stored [Ca^2+^]_mt_ in Cdk5^*−/−*^ MEFs compared with wt. MEFs obtained from two different sets of wt and Cdk5^*−/−*^ embryos were used at passage 2–7. Values are means ± SEM from the four independent experiments. *p < 0.05 by unpaired Student’s *t* test.

A rise in mitochondrial Ca^2+^ level, [Ca^2+^]_mt_, has been shown to be a critical regulator of mPTP opening [41]. Thus, we sought to examine potential alteration in [Ca^2+^]_mt_ in *Cdk5*^*−/−*^ MEFs by tracing cytoplasmic Ca^2+^ level, [Ca^2+^]_cyt_, following the addition of the protonophore and oxidative phosphorylation uncoupler, FCCP. FCCP causes depolarization or collapse of the mitochondrial membrane potential, resulting in mPTP opening and release of Ca^2+^ from the mitochondria [42]. Therefore, the increase in cytoplasmic Ca^2+^ level following FCCP treatment in wt as well as *Cdk5*^*−/−*^ MEFs corresponds to [Ca^2+^]_mt_. To proceed with [Ca^2+^]_mt_ measurement, wt and *Cdk5*^*−/−*^ MEFs loaded with the cell-permeable intracellular calcium indicator, Fluo-4-AM, were subjected to single cell Ca^2+^ imaging before and after FCCP treatment. As shown in Fig. 2a, treatment with FCCP caused a greater wave of increase in [Ca^2+^]_cyt_ in *Cdk5*^*−/−*^ MEFs than in wt, indicating increased [Ca^2+^]_mt_ in *Cdk5*^*−/−*^ MEFs compared with wt. Quantitative analyses revealed a 58% increase (*p* < 0.05) in peak amplitude (Fig. 2b) and a twofold increase (*p* < 0.05) in area under the curve (Fig. 2c) in *Cdk5*^*−/−*^ MEFs compared with wt further indicating increased [Ca^2+^]_mt_ in *Cdk5*^*−/−*^ MEFs.

### Cdk5 is a mitochondria-associated ER membrane (MAM) protein, which when lost induces ER-mitochondria tethering

**Fig. 3:**
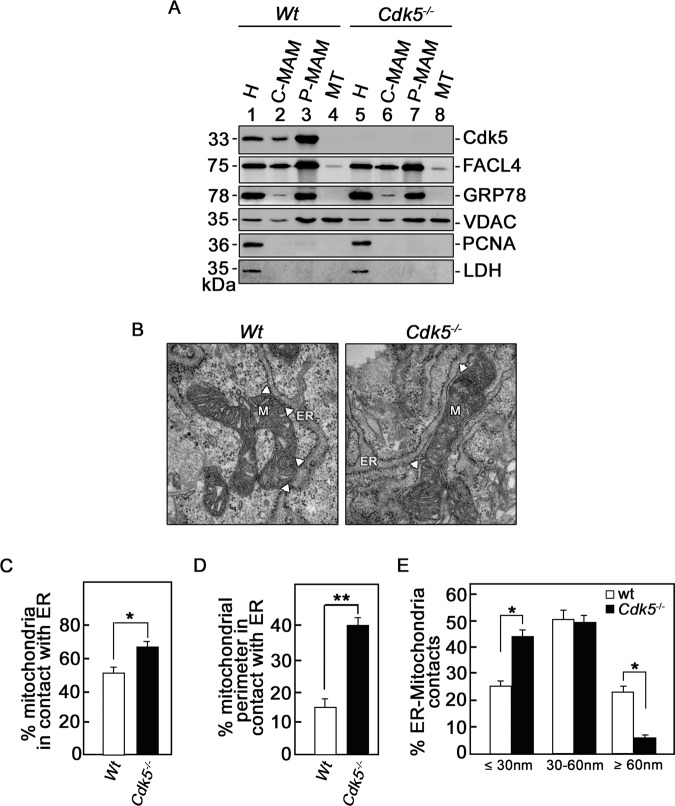
Cdk5 localizes in MAMs and loss of Cdk5 causes increased number and length of ER-mitochondria contact sites and reduced contact distance between these two organelles. **a** Immunoblot analysis of purified MAM and mitochondria in wt and *Cdk5*^*−/−*^ MEFs. 50 μl of homogenate (H), crude MAM (C-MAM), purified MAM (P-MAM), and purified mitochondria (MT) were loaded. For subcellular markers, fatty acid-CoA ligase, long-chain (FACL4) and glucose-regulated protein 78 kDa (GRP78) were used for ER-MAM; VDAC for mitochondria-MAM; proliferating cell nuclear antigen (PCNA) for nucleus; and lactate dehydrogenase (LDH) for cytoplasm. Blots shown represent data from one of three independent experiments (*n* = 3) showing similar results. **b**–**e** Transmission electron microscopy (TEM) of ER-mitochondria tethering in wt and *Cdk5*^*−/−*^ MEFs. Images were acquired using a Hitachi H7650 TEM. Scale bar = 100 nm. ER: endoplasmic reticulum; M: mitochondria. Arrowheads are directed at ER-mitochondria contact sites. For the morphometric analysis in **c**–**e**, a total of six TEM grids (3.05 mm discs) prepared from two embeddings per genotype were analyzed: four grids from embedding 1 and two grids from embedding 2. Mitochondrial counts and mitochondria-ER contact measurements were performed in groups of two grids (*n* = 3, where replicates 1 and 2 originate from the four grids from embedding 1 and replicate 3 originates from the two grids from embedding 2. In **c**, all observed mitochondria in wt and *Cdk5*^*−/−*^ MEFs, irrespective of the number of cells, were counted and the percentage of mitochondria in contact with ER was calculated. In **d**, the mitochondrial perimeter in contact with ER was measured and the percentage of the total individual mitochondrial perimeter in contact with ER was calculated. In **e**, distances between ER and mitochondria in contact sites were measured and the percentage of the total contact sites with different ranges of ER-mitochondria distances were calculated. In **d** and **e**, a total of at least 40 ER-mitochondria contacts, irrespective of the number of cells, were analyzed per two TEM grids (i.e., at least 120 ER-mitochondria contacts were analyzed per genotype).). Embeddings were from different cultures of MEFs. Values are means ± SEM; *n* = 3; *p < 0.05 and **p < 0.01 by unpaired Student’s *t* test.

The ER is the major intracellular Ca^2+^ store, and the interface between the ER and the mitochondria, named mitochondria-associated ER membrane (MAM), contains channels for calcium transfer [21]. As shown in Fig. 3a, Cdk5 exists in MAMs purified from wt MEFs. Cdk5 as a MAM protein was supported by co-fractionation with the ER-MAM markers, fatty acid-CoA ligase long-chain (FACL4) and glucose-regulated protein 78 kDa (GRP78), and the mitochondria-MAM marker, voltage-dependent anion channel (VDAC). Purity of the isolated MAMs was further assessed by the lack of the nuclear and cytoplastic markers, proliferating cell nuclear antigen (PCNA) and lactate dehydrogenase (LDH), respectively.

ER-mitochondria tethering is essential for the formation of ER-mitochondria contact sites and subsequent Ca^2+^ transfer from the ER to the mitochondria [43, 44]. To investigate the possibility that ER-mitochondria tethering and contact sites are altered in *Cdk5*^*−/−*^ MEFs, causing an increase in [Ca^2+^]_mt_, ultrathin sections of wt and *Cdk5*^*−/−*^ MEFs were subjected to TEM (Fig. 3b) as described in the Materials and Methods section. Since the number, length and distance between the ER-mitochondria contact sites have been shown to influence ER Ca^2+^ transfer to the mitochondria [43, 45, 46], we analyzed these parameters in wt and *Cdk5*^*−/−*^ MEFs. As shown in Fig. 3c, the percentage of mitochondria that is in contact with the ER is higher (*p* < 0.05) in *Cdk5*^*−/−*^ MEFs than in wt (67% vs 52 %). Figure 3d shows that the mitochondrial perimeter that is in contact with the ER in *Cdk5*^*−/−*^ MEFs is also greater (*p* < 0.01) in *Cdk5*^*−/−*^ MEFs than in wt (40% vs 15%). In addition, as shown in Fig. 3e, the percentage of ER-mitochondria contact sites with a distance of <30 nm is higher (*p* < 0.05) in *Cdk5*^*−/−*^ MEFs compared with wt (44% vs 25%).

### Loss of Cdk5 causes increased mitochondrial Ca^2+^ uptake from the ER upon stimulation with ATP

**Fig. 4:**
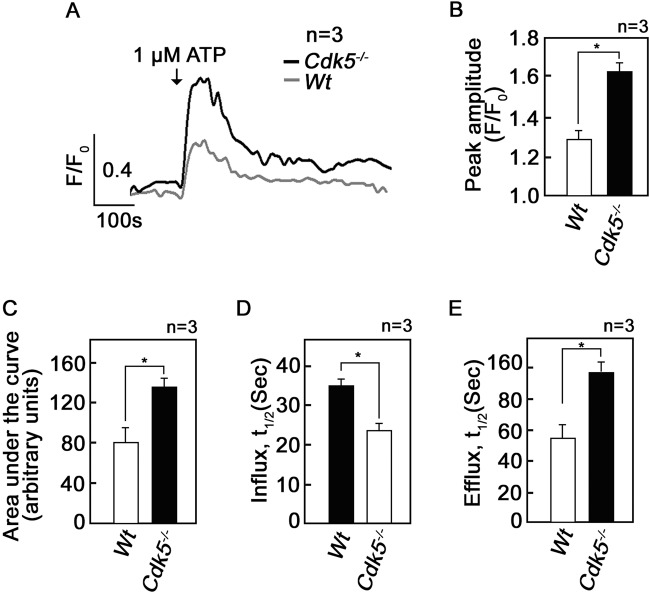
Stimulation with ATP causes greater mitochondrial Ca^2+^ uptake from the ER in *Cdk5*^*−/−*^ MEFs compared to wt. **a** Wt and *Cdk5*^*−/−*^ MEFs loaded with Rhod-2 AM (5 µM) for 30 min in Ca^2+^ free buffer were analysed by single cell Ca^2+^ imaging. ATP (1 µM) was added where indicated. Data show mean values of Ca^2+^ signals from 15 randomly selected cells and represent one of three independent experiments showing a similar results. Analysis of results from the three independent experiments (*n* = 3), each with data from 10–15 randomly selected cells from each genotype, showed that compared to wt, *Cdk5*^*−/−*^ MEFs exhibited: **b** higher peak amplitude (*F*/*F*_0_), corresponding to greater Ca^2+^ influx into the mitochondria; **c** increased integrated Ca^2+^ signals (area under the curve from 120 to 600 s), which also indicates elevated Ca^2+^ entry into the mitochondria; **d** reduced time of Ca^2+^ influx into mitochondria, indicating faster rate of influx; and **e** increased time of efflux, indicating slower rate of efflux. Values are means ± SEM. *p < 0.05 by unpaired Student’s *t* test.

Our next approach was to examine a potential difference in mitochondrial Ca^2+^ uptake from the ER in wt and *Cdk5*^*−/−*^ MEFs. To do so, cells in Ca^2+^-free buffer and pre-loaded with a mitochondrial Ca^2+^ probe, Rhod-2 AM [47], were treated with ATP, which binds cell surface purinergic receptors [48] and IP3R [49], which both stimulate IP3-evoked ER Ca^2+^ release [49]. As shown in Fig. 4a, treatment with ATP caused a greater wave of increase in [Ca^2+^]_mt_ in *Cdk5*^*−/−*^ MEFs than in wt. Quantitative analyses showed a 26% increase (*p* < 0.05) in peak amplitude (Fig. 4b), and a 69% increase in integrated Ca^2+^ signal (Fig. 4c) in *Cdk5*^*−/−*^ MEFs compared to wt. In addition, the rate of Ca^2+^ influx into the mitochondria was 33% faster in *Cdk5*^*−/−*^ MEFs compared to wt (Fig. 4d) while the rate of Ca^2+^ extrusion from the mitochondria was slower by 75% in *Cdk5*^*−/−*^ MEFs compared to wt (Fig. 4e). Together, our data indicate that *Cdk5*^*−/−*^ MEFs have increased and prolonged ATP-induced mitochondrial Ca^2+^ uptake from the ER compared to wt.

### mPTP opening due to Cdk5 loss is blocked by inhibition of ER Ca^2+^ release or mitochondrial calcium uptake

**Fig. 5:**
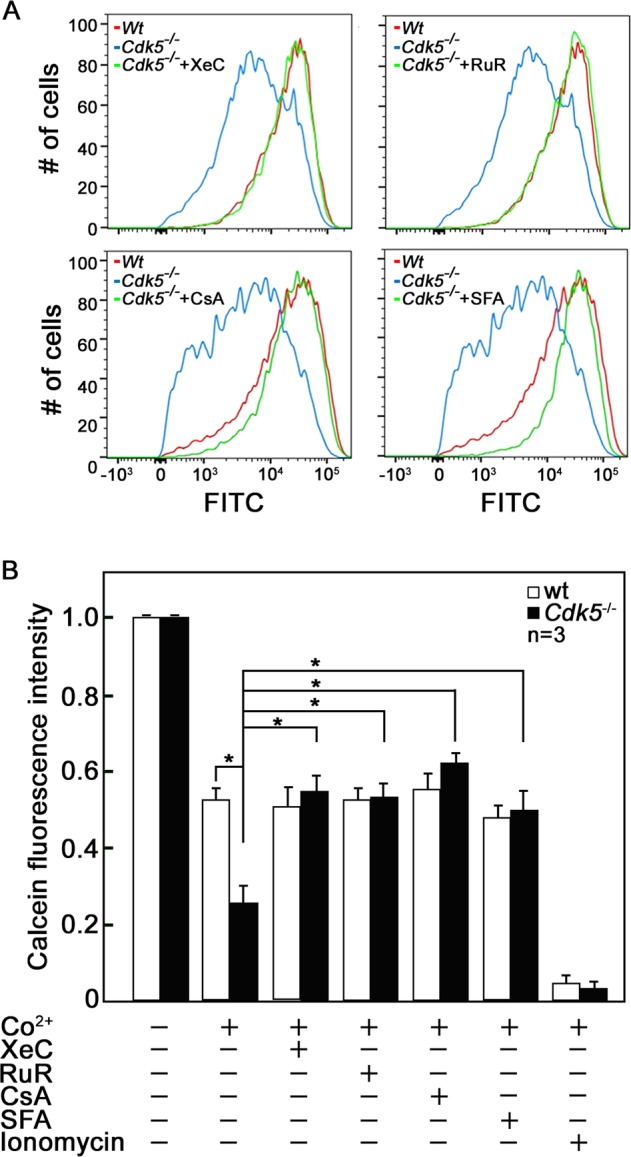
Inhibition of IP3R by xestospongin C (XeC) or MCU by ruthenium red (RuR) reverses the opening of mPTP in *Cdk5*^*−/−*^ MEFs. **a** Wt and *Cdk5*^*−/−*^ MEFs pretreated with or without XeC (3 µM) or RuR (3 µM) were loaded with calcein-AM, then treated with or without CoCl_2_. Cell were then analysed by flow cytometry. Cells treated with the potent mPTP desensitizers, cyclosporin A (CsA, 1 µM) or sanglifehrin A (SFA, 2 µM), were used as positive controls. **b** Quantitative analysis of the relative calcein fluorescence in wt and *Cdk5*^*−/−*^ MEFs in the presence or absence of the treatments indicated above. Untreated cells and cells treated with ionomycin served as controls. Values are means ± SEM from three (*n* = 3) independent experiments. *p < 0.05 by unpaired Student’s *t* test.

We then investigated whether inhibition of either ER Ca^2+^ release with the potent IP3R inhibitor, XeC [50, 51], or mitochondrial calcium uptake with the potent MCU inhibitor, RuR [52], followed by calcein-AM loading and subsequent treatment with CoCl_2_ would show blockade of increased mPTP opening in *Cdk5*^*−/−*^ MEFs. Consistent with our data in Fig. 1, flow cytometry analyses shown in Fig. 5, a, b demonstrate that *Cdk5*^*−/−*^ MEFs exhibited less calcein fluorescence intensity compared to wt, indicating greater Co^2+^ quenching of mitochondrial calcein fluorescence and thus increased mPTP opening in *Cdk5*^*−/−*^ MEFs compared to wt. However, treatment with XeC or RuR increased the calcein fluorescence intensity in *Cdk5*^*−/−*^ MEFs to a level equivalent to that of wt. Treatment with the potent mPTP desensitizers, CsA [53] or SFA [54] also completely prevented mPTP opening in *Cdk5*^*−/−*^ MEFs. Cells untreated with CoCl_2_ and cells treated with ionomycin served as controls. Altogether, we demonstrate that inhibition of either ER Ca^2+^ release or mitochondrial calcium uptake prevents mPTP opening in *Cdk5*^*−/−*^ MEFs, further indicating that mPTP opening due to loss of Cdk5 results from increased ER Ca^2+^ transfer to mitochondria.

## Discussion

**Fig. 6:**
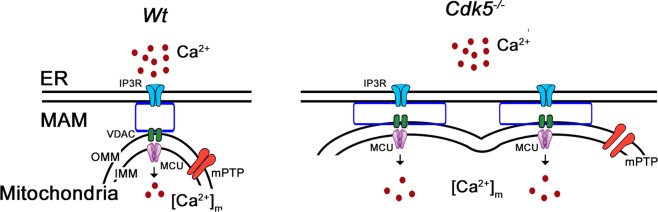
Proposed model illustrating mPTP opening in wt and *Cdk5*^*−/−*^ MEFs. Loss of Cdk5 that localizes in MAM causes reduced distance between ER and mitochondria, and increased number and length of ER-mitochondria contact sites, causing increased Ca^2+^ transfer from the ER to the mitochondria, which leads to increased [Ca^2+^]_mt_ and subsequently, mPTP opening.

We now know that loss of Cdk5 in breast cancer cells causes increased mPTP opening that is associated with mitochondrial depolarization, increased ROS levels, mitochondrial fragmentation and apoptosis [6]. Furthermore, we determined that the mPTP-mediated apoptotic pathway in breast cancer cells is coupled with an increase in both intracellular [Ca^2+^] level and calcineurin activity [6]. In the current study, we identify a novel mechanism by which loss of Cdk5 promotes mPTP opening. Figure 6 illustrates that Cdk5 loss in *Cdk5*^*−/−*^ MEFs causes an increase in number and length of ER-mitochondrial contact sites as well as a decrease in gaps between these contact sites. These changes promote an increase in mitochondrial Ca^2+^ uptake from the ER, causing increased [Ca^2+^]_mt_ and subsequent increase in mPTP opening upon loss of Cdk5.

mPTP is a calcium-dependent and CsA-sensitive high-conductance channel. mPTP opening induces permeability of the IMM due to the opening of its pore forming proteins. The components of mPTP and the pore-forming proteins remain unresolved. Nonetheless, several studies implicate physiological roles of mPTP in Ca^2+^ buffering, energy metabolism, and mitochondrial homeostasis. Our group [6] and others [33, 34, 55, 56] also showed that increased or prolonged mPTP opening results in mitochondrial depolarization, increased ROS generation, inhibition of ATP synthesis, and release of apoptosis-inducing mitochondrial proteins such as cytochrome C and apoptosis inducing factor, causing activation of the caspase-mediated apoptotic pathway. We observed these mPTP opening-induced characteristics in breast cancer cells lacking Cdk5 but *Cdk5*^*−/−*^ MEFs also exhibit increased mPTP opening, which is reversed by mPTP desensitizers such as CsA and SFA and show increased [Ca^2+^]_mt_ level that has been shown to induce mPTP opening. As with breast cancer cells depleted of Cdk5 by siRNA, we determined here that *Cdk5*^*−/−*^ MEFs exhibit increased mPTP opening that is reversed by the mPTP desensitizers, CsA and SFA, and show increased [Ca^2+^]_mt_ level that induces mPTP opening. It is interesting, however, that while loss of Cdk5 in breast cancer cells promote apoptosis, we observed that *Cdk5*^*−/−*^ MEFs show increased proliferation (data not shown). This was unexpected but supports differential physiology and roles of Cdk5 in normal and cancer cells, and calls for more comprehensive investigations on the function of Cdk5 in mitochondrial calcium dynamics in normal and disease conditions.

MAM, a proteinaceous link between the ER and mitochondria, is important for calcium and metabolite transfer between these organelles. In this study, we show that Cdk5 is enriched in pure MAM fractions, a finding that is consistent with the mass spectrometry analysis by Poston et al., identifying, with high confidence, Cdk5 as one of the MAM proteins in mouse brain [57]. For effective calcium transfer, MCU channels require close ER-mitochondria apposition that is influenced by the ER-mitochondria contact site number, length and distance. The ER-mitochondria contact sites allow the rapid transfer of large amounts of Ca^2+^ from the ER to the mitochondria upon opening of the IP_3_-gated channels [23, 24, 58]. The distance from the OMM to the rough and smooth ER was determined to be 10–30 nm and 9–16 nm respectively [45, 59], and about 5–20% of the mitochondrial surface is closely apposed to the ER [26, 27]. Our studies show that in wt cells, the ER is juxtaposed to 15% of the mitochondrial surface whereas loss of Cdk5 increases the ER-mitochondrial juxtaposition to about 40%. Similarly, the percentage of ER-mitochondria contacts within ≤30 nm is higher in the absence of Cdk5. Since disruption in ER-mitochondria tethering alters Ca^2+^ transfer between the ER and the mitochondria [60], it is not surprising that we observed increased ATP-induced mitochondrial calcium uptake in cells lacking Cdk5. Similarly, prolonged extrusion of mitochondrial Ca^2+^ in the absence of Cdk5 is expected to contribute to [Ca^2+^]_mt_ accumulation and subsequent mPTP opening. Nevertheless, our observations indicate that Cdk5 in MAM serves to control ER-mitochondrial tethering, and loss of Cdk5 causes an upsurge in mitochondrial calcium uptake by increasing ER-mitochondrial tethering. While studies are underway to further characterize the specific role of MAM Cdk5 in ER-mitochondria tethering, our finding that inhibition of either ER Ca^2+^ release with XeC or mitochondrial calcium uptake with RuR blocks the increase in mPTP opening induced by Cdk5 loss suggests that elevated mitochondrial calcium influx underlies the increase in mPTP opening in the absence of Cdk5. Altogether, our studies demonstrate a novel mechanism whereby Cdk5 controls mPTP opening by regulating the formation of ER-mitochondria contact site structures and subsequently, calcium transfer from the ER to the mitochondria and [Ca^2+^]_mt_ level.

## Materials and methods

### Materials

Dulbecco’s modified eagle’s medium (DMEM), heat-inactivated fetal bovine serum (FBS), EDTA-Trypsin, antibiotic-antimycotic, Calcein AM, Fluo-4 AM and Rhod-2 AM were from Life Technologies Inc. The protease inhibitor cocktail, Cyclosporine A (CsA), Ruthenium Red (RuR), ATP, carbonyl cyanide-p-trifluoromethoxyphenylhydrazone (FCCP) and Xestospongin C (XeC) were from Sigma. Antibodies to Cdk5 (C-8), FACL-4 (F-4), VDAC1 (B-6), GRP78 (A-10), PCNA (PC-10) and LDH-A (E-9) were from Santa Cruz Biotech. Sanglifehrin A (SFA) was a gift from Novartis (Switzerland).

### Primary MEF isolation and culture

Primary MEFs were isolated from E13.5 wt and *Cdk5*^*−/−*^ mouse embryos following the protocol described by Durkin et al. [35] which was based on the method by Todaro and Green [36] and subsequent modifications of the technique by Coats et al. [37]. Embryos were from *Cdk5*^+/−^ pregnant female mice crossed with *Cdk5*^+/−^ male mice. Embryos were washed with (phosphate-buffered saline) PBS, decapitated and eviscerated then washed again with PBS. Embryos were minced with sterile forceps and placed in 3–5 ml of 0.05% trypsin-EDTA, pipetted up and down to get cells into suspension and incubated at 37 °C for 5 min. Cell suspensions were transferred to new tubes containing MEF medium (DMEM-high glucose supplemented with 10% FBS, 1% penicillin-streptomycin and 2 mM GlutaMAX) then centrifuged at 1,000 rpm for 5 min. Cell pellets were resuspended in fresh media and plated in 10 cm cell culture dishes. Primary MEFs were cultured in DMEM supplemented with 10% FBS and 100 U/ml each of penicillin and streptomycin under hypoxic condition (5% O_2_ and 5% CO_2_ incubator). All experiments were performed in passage P2-P7 MEFs.

### Transmission electron microscopy

TEM analysis was performed following fixation, dehydration, infiltration, and embedding of wt and *Cdk5*^*−/−*^ MEFs in situ. Ultra-thin (~60 nm) sections were cut and stained with 2% uranyl acetate and lead citrate and observed under a Hitachi H7650 TEM at the University of Calgary’s Microscopy and Imaging Facility. Images were acquired through an AMT 16000 CCD mounted on the microscope.

### MAM and mitochondria isolation

Crude and pure MAMs, and mitochondria were isolated using Percoll density gradient centrifugation as described previously [38]. Briefly, trypsinized MEFs were homogenized in 10 ml of IB_cells_-1 buffer containing 225 mM mannitol, 75 mM sucrose, 0.1 mM EGTA, and 30 mM Tris–HCl (pH 7.4). Homogenates (H) were centrifuged at 600 × g for 5 min at 4 °C. The resulting supernatants were subjected to further centrifugation at 7000 × *g* for 10 min at 4 °C. Pellets were resuspended in 10 ml of IB_cells_-2 buffer containing 225 mM mannitol, 75 mM sucrose and 30 mM Tris–HCl (pH 7.4) followed by centrifugation at 7000 × *g* for 10 min at 4 °C. Pellets containing MAM and mitochondria were resuspended again in 10 ml of IB_cells_-2 buffer and centrifuged at 10,000 × *g* for 10 min at 4 °C. The resulting pellets were resuspended in 2 ml of mitochondria resuspending buffer (MRB: 250 mM mannitol, 0.5 mM EGTA and 5 mM HEPES, pH 7.4). The crude mitochondria were then layered with 8 ml of Percoll medium [225 mM mannitol, 25 mM HEPES (pH 7.4), 1 mM EGTA and 30% Percoll] and 4 ml of MRB then centrifuged at 95,000 × *g* for 30 min at 4 °C. Pure mitochondria (MT) settles at the bottom of the tube while the MAM fraction appears as a white band above the MT fraction. The MAM and MT fractions were each resuspended in 14 ml of MRB buffer and centrifuged at 6300 × *g* for 10 min at 4 °C. The resulting supernatants (~14 ml) of the MAM fraction, designated as crude MAM (C-MAM), were further subjected to centrifugation at 100,000 × *g* for 1 hr. Pellets containing pure MAM (P-MAM) or pure MT were each resuspended in 50 µl of MRB buffer.

### Western blotting

Isolated subcellular fractions (50 µl) were loaded in 12.5% SDS polyacrylamide gels and transferred onto nitrocellulose membranes. Membranes were blocked in 5% skimmed milk then incubated with the indicated primary antibody (1:1000 dilution) at 4 °C overnight. After washing with Tris-buffered saline (TBS) + Tween-20 (TBST) (50 mM Tris–HCl, pH 7.6, 0.1% Tween-20, 0.8% NaCl), membranes were incubated with horseradish peroxidase-conjugated secondary antibody (1:10,000 dilution) for 1 h. Immunoreactive bands were detected using ECL reagent (Amersham).

### mPTP opening assay

Calcein fluorescence was measured using the Image-IT live mitochondria permeability transition pore assay kit (Thermo Fisher Sci.) as per the manufacturer’s protocol. Briefly, cells seeded on a four-chamber cover glass (Lab-Tek) were loaded with calcein AM (1 µM), MitoTracker Red (200 nM), treated with or without CoCl_2_ (1 mM) in Hank’s balanced salt solution (HBSS), and incubated for 15 min at 37 °C under 5% CO_2_. Cells were then washed with HBSS buffer and images were taken under an Olympus 1 × 71 fluorescence microscope using a ×10 objective with or without the ×1.6 magnification changer.

For flow cytometry, cells (1 × 10^4^) were harvested using Trypsin-EDTA and pre-incubated with CsA (1 µM), SFA (2 µM), XeC (3 µM) or RuR (3 µM) for 1 h then stained as indicated above. Cells were then washed with HBSS, resuspended in ice-cold PBS and analyzed by flow cytometry using a fluorescein isothiocyanate filter (530 nm) for measuring calcein fluorescence.

### Calcium measurement

For cytoplasmic Ca^2+^ measurements, MEFs seeded on 3.5 cm glass bottom petri dishes (80% confluency) were loaded with Fluo-4 AM (5 µM) in HBSS (with 1.26 mM calcium) for 30 min at RT. Cells were washed three times with KRH buffer (125 mM NaCl, 5 mM KCl, 1.2 mM MgCl_2_, 25 mM HEPES, 6 mM glucose) then subjected to single cell calcium imaging using a Leica TCS SP8 confocal microscope (×20 objective). Fluorescence signals were quantified as ratio (*F*/*F*_0_) of the fluorescence after addition of FCCP, relative to the basal fluorescence (*F*_0_) before stimulation.

For mitochondrial Ca^2+^ measurements, MEF cells seeded on 3.5 cm glass bottom petri dishes (80% confluency) were loaded with Rhod-2 AM (5 µM) in KRH buffer (125 mM NaCl, 5 mM KCl, 25 mM HEPES, 6 mM glucose) for 30 min at RT. Cells washed three times with KRH buffer were subjected to single cell calcium imaging using a Zeiss LSM 510 Meta laser scanning confocal microscope (×20 objective). Fluorescence signals were quantified as ratio (*F*/*F*_0_) of the fluorescence after addition of ATP, relative to the basal fluorescence (*F*_0_) before stimulation.

### Statistical analysis

Student’s *t* test (unpaired, two-sided) was used. Significance was set at *p* < 0.05.
